# Genomic and transcriptional analysis of protein heterogeneity of the honeybee venom allergen Api m 6

**DOI:** 10.1111/j.1365-2583.2006.00669.x

**Published:** 2006-10

**Authors:** N Peiren, D C de Graaf, J D Evans, F J Jacobs

**Affiliations:** Laboratory of Zoophysiology, Ghent University Ghent, Belgium; * USDA-ARS Bee Research Laboratory Beltsville, MD, USA

**Keywords:** allelic variation, allergen, Api m 6, bee venom, signal peptide

## Abstract

Several components of honeybee venom are known to cause allergenic responses in humans and other vertebrates. One such component, the minor allergen Api m 6, has been known to show amino acid variation but the genetic mechanism for this variation is unknown. Here we show that Api m 6 is derived from a single locus, and that substantial protein-level variation has a simple genome-level cause, without the need to invoke multiple loci or alternatively spliced exons. Api m 6 sits near a misassembled section of the honeybee genome sequence, and we propose that a substantial number of indels at and near Api m 6 might be the root cause of this misassembly. We suggest that genes such as Api m 6 with coding-region or untranslated region indels might have had a strong effect on the assembly of this draft of the honeybee genome.

## Introduction

Dangerous allergenic responses can be caused in humans by components of the venom of stinging social insects (Hoffman, 2003). A major research goal remains to predict which fraction of the human population will be likely to react adversely to specific venom components received through incidental or occupation-related stinging. Further, an understanding of the components of venoms that induce allergic responses can be used to better tune therapeutic treatments of allergic responses. Allergens from insects and other sources are designated with the terms ‘major’ or ‘minor’ depending on whether more or less than 50% of the hypersensitive patients develops a specific IgE response against a given allergen (Larsen & Lowenstein, 1996). Honeybee venom contains the major allergens Api m 1 (phospholipase A2), Api m 2 (hyaluronidase), Api m 3 (acid phosphatase), Api m 4 (melittin) and Api m 7 (CUB serine protease), along with at least one minor allergen, Api m 6, which shows 42% IgE responsiveness (Hoffman, 2006).

Here we describe the genomic basis for observed protein-level heterogeneity found for Api m 6. Api m 6 migrates as an 8-kDa band in sodium dodecyl sulphate–polyacrylamide gel electrophoresis and its amino acid sequence was determined on high-performance liquid chromatography-purified preparations (Kettner *et al*., 2001). It exists as four isoforms of 7190, 7400, 7598 and 7808 Da, respectively, differing in their primary structure at the amino and carboxy terminus by a maximum of six amino acids (Kettner *et al*., 2001). Allergen heterogeneity can be due to allelic variation at a single allergen gene (Gao *et al*., 2005), the occurrence of multiple genes of allergens encoding highly homologous proteins (Piersma *et al*., 2005), or by alternative splicing of a single transcript (Mykles *et al*., 1998). Some bee venom components are already known to have a rather peculiar gene organization. Indeed, the precursors of bee venom apamin and MCD peptide are encoded by two genes in tandem, which share the same 3′-exon (Gmachl & Kreil, 1995). Here we use new genome-level data for honeybees to determine the cause behind multiple isoforms of Api m 6. We present the complete cDNA sequences for two Api m 6 variants. We then use the latest honeybee assembly to show that these variants arise from a single polymorphic locus. Interestingly, high sequence-level variation at and around this locus appears to be responsible for a break in the honeybee genome assembly.

## Results and discussion

### Cloning and sequencing of cDNA fragments encoding Api m 6

5′-Rapid amplification of cDNA ends (5′-RACE) using gene-specific primers resulted in an amplicon of approximately 350 bp in size that was cloned in the pCR®4 vector. Transformation to chemically competent TOP-10 *Escherichia coli* yielded a large number of transformants, 10 of which were retained for further analysis. DNA sequences were found to be identical (represented by *clones 5.1*) and consisted of a putative 5′ untranslated region (UTR) and a coding region for which the predicted protein was identical to that known for Api m 6. 3′-RACE resulted in a fragment of approximately the same size, which was again cloned and sequenced. Here the sequenced clones showed two transcript variants, represented by the inserts of *clone 3.3* and *clone 3.9*. Subsequently, we amplified the complete Api m 6 transcript from a venom gland cDNA preparation using primers designed, respectively, at the extreme 5′- and 3′-ends of *clone 5.1* and *clone 3.9*. Sequencing of the corresponding 528 bp fragment revealed two additional minor transcript differences, this time at the 5′-end, 38 and 117 bp upstream of the forward primer, when compared with the insert of *clone 5.1*. This cDNA sequence was deposited to GenBank (accession number DQ384991), as was a sequence assembly of the inserts of *clones 5.1* and *3.9* (accession number DQ384990). Because the latter matched perfectly well the latest honeybee genome assembly (see further), it was named variant 1, whereas DQ384991 became variant 2.

### Deduced amino acid sequence and protein heterogeneity

**Figure 1:**
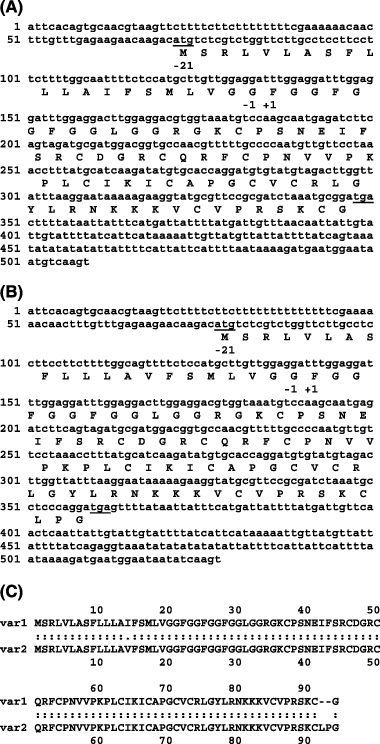
Nucleotide and deduced amino acid sequences of Api m 6 transcript variant 1 (A) and 2 (B). The numbers on the left denote the nucleotide numbers. The start and stop codon are underlined. Residues −1 to −21 are from the signal peptide. Residue +1 denotes the first amino acid of the mature protein. In (C) the alignment of the deduced amino acid sequences of the two Api m 6 transcript variants is given.

The deduced amino acid sequences of both transcripts are given in Fig. 1. At the protein level differences were noticed at position 14 (Val against Ile) and at the carboxy terminal region, with two additional residues Leu and Pro. This corresponds with the differences found at the C-terminal ends of the four Api m 6 variants described by Kettner *et al*. (2001): Api m 6.01 and Api m 6.03 having the same two additional residues, in contrast to the variants Api m 6.02 and Api m 6.04. Further we noticed that the deduced amino acid sequences were 21 residues longer at the N-terminus when compared with the earlier described variants Api m 6.03 and Api m 6.04 (Kettner *et al*., 2001). However, based on an *in silico* SigP-NN prediction (Bendtsen *et al*., 2004), a signal peptide cleavage site was identified between position 21 and 22, resulting in a mature protein that starts at exactly the same residue. Depending on the cDNA variant that was translated this mature protein will correspond to Api m 6.03 or Api m 6.04. The other two variants described by Kettner *et al*. (2001) lack the first four N-terminal amino acids Phe-Gly-Gly-Phe of the mature protein. We have found no indication that this was the result of a new transcript or alternative splicing variant. Nor could we evidence that the mature proteins of these variants are shortened by enzymatic activity similar to step-wise cleavage of the pro part from promelittin by dipeptidylpeptidase IV (Kreil *et al*., 1980). Further we notice that amino acid replacement at position 14 in the deduced amino acid sequence is located in the signal peptide and has no consequences for the mature allergen.

### Sequence homology and conserved domains

Conserved domain search (Marchler-Bauer & Bryant, 2004) of the deduced amino acid sequence from transcript variant 1 revealed a trypsin inhibitor like cysteine rich (TIL) domain from residue 37–91 (score: 37.3; *E*-value: 5e-04). This family contains trypsin inhibitors as well as a domain found in many extracellular proteins. The domain typically contains 10 cysteine residues that form five disulphide bonds in the combination 1–7, 2–6, 3–5, 4–10 and 8–9. The assumption that Api m 6 represents a trypsin inhibitor was already made by Banks & Shipolini (1986), although the protein was at that time hardly characterized and certainly not yet named as such. In fact, they described two peptides H1 (17 residues) and H3 (35 residues), which later were found to correspond with the N-terminal ends of Kettner's isoforms of Api m 6, differing from each other only in that one lacks the first four residues. The amino acid compositions were similar to a protease inhibitor that was earlier purified from bee venom by Shkenderov (1973). Although no proteolytic activity could be assigned to the peptides H1 and H3, it appears now that Api m 6 has a molecular weight quite near that of Shkenderov's protease inhibitor, i.e. 9000 Da.

### Noncoding sequence variation and the genome assembly

**Figure 2:**
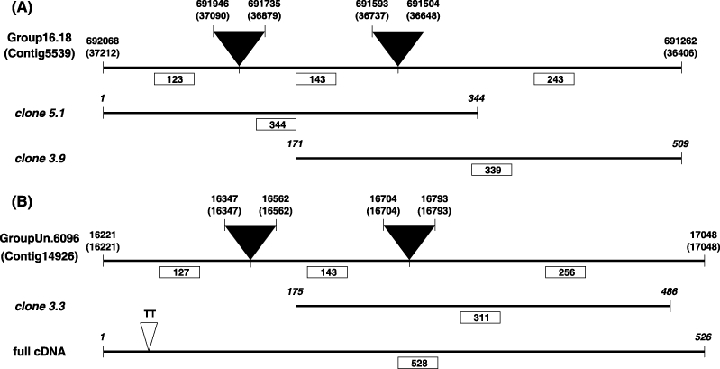
Alignment of two *in silico* spliced honeybee genome sequences with different cDNA fragments of the bee venom allergen Api m 6. (A) Api m 6 was present in mapped scaffold 16.18 from genome assembly 4.0 (positions marked above the bar, along with coordinates for the corresponding Contig5339). Api m 6 is characterized by two introns (depicted by black triangles; splicing sites given on top) and this haplotype of the genome assembly showed a perfect sequence-level match with cloned 5′- (*clone 5.1*) and 3′-RACE fragments (*clone 3.9*). The predicted protein from this sequence is identical to the described Api m 6 variant 1. (B) A second transcript variant was found by cDNA sequencing (full cDNA). This transcript has a nearly identical match to unmapped scaffold GroupUn.6097 (Contig14926), the only difference being a TT-insertion mutation (depicted by a white triangle) in the 5′-UTR. The 3′-end of transcript variant 2 corresponds also to another cloned 3′-RACE fragment (*clone 3.3*). Fragment lengths (in bp) are given below the bar and in boxes.

A BLASTN search (Altschul *et al*., 1997) of the complete cDNA against honeybee genome assembly 4.0 (Amel_4.0–20061003) revealed two hits with very high scores/*E*-values: scaffold 16.18 (Contig5539), score: 486; *E*-value: e-136 and unmapped scaffold GroupUn.6096 (Contig14926), score: 371; *E*-value: e-101. DNA sequence comparison demonstrated a 100% match between Contig5539 (between position 37212 and 36406, introns excluded) and an assembly of the RACE fragments from *clone 5.1* and *clone 3.9* (Api m 6 variant 1, Fig. 2A). On the other hand, Contig14926 (between position 16221 and 17048, introns excluded) was identical to the coding region of the full size Api m 6 transcript and the insert of *clone 3.3* (Api m 6 variant 2, Fig. 2B). However, the UTR of the full size cDNA shows an indel of two consecutive thymines, found upstream from position 41 in DQ384991. There are also eight indels (at four locations) and one G to A transition in the 3′ UTR of variant 1 when aligned with variant 2. These results demonstrate the existence of several different transcript variants of the bee venom allergen Api m 6, originating from genome-level variation at a single locus.

Api m 6 shows a substantial amount of sequence variation (nearly all haplotypes sequenced to date differ in at least one place) as well as sections of repetitive simple sequences (monobasic A or T runs, as well as an AT dinucleotide repeat; Fig. 1). It is conceivable that this variation was the root cause for misassembly at this section of the draft honeybee genome sequence. First, it is evident that assembly scaffolds 16.18 and GroupUn.6095 are homologous, and that GroupUn.6095 spans the gap between assembly scaffolds 16.18 and 16.19. These genome sections apparently assembled separately because of sequence variation found in the two haplotypes sequenced for the Honeybee Genome Project. This effect is probably widespread in assemblies of this and other draft genome sequences. In fact, this effect is seen further down chromosome 16 (scaffolds 16.11 and 16.12), where the hypervariable immune effector apidaecin (Casteels *et al*., 1994) has apparently disrupted the genome assembly. An understanding of allelic variation at specific genes might help unite unassembled parts of the bee genome. As a corollary, misassembled sections of the bee genome might indicate biologically important variation in genes adjacent to these gaps.

## Experimental procedures

### Bee venom glands

Honeybees (*Apis mellifera carnica*) were all taken at the hive entrance of a single colony from the apiary of the Ghent University (Belgium). The venom glands of 320 bees were dissected under anaesthesia by chilling. First, the whole sting apparatus was removed from the abdomen and submerged in RNALater® (Ambion, Austin, TX, USA). Subsequently, the glands were separated from the reservoir and collected all together in 100 µl fresh solution. Homogenization and mRNA isolation was done using the Micro-FastTrack™ 2.0 Kit (Invitrogen, Carlsbad, CA, USA) following the protocol for fresh and frozen tissue. After spectrophotometric yield determination, mRNA was stored in elution buffer at −80 °C until ready for use.

### cDNA preparation and primer development

Venom gland cDNA was prepared using AMV reverse transcriptase and the oligo dT primer from the cDNA Cycle® Kit (Invitrogen) according to the manufacturer's instructions. To find the corresponding genome sequence for primer development, the Api m 6 amino acid sequence (P83563) was Blast-searched against an early release of the honeybee genome (Amel_1.2) at http://www.hgsc.bcm.tmc.edu/projects/honeybee. The target domain was located on group 16.4 and extended from position 258118–258415 (total length of the group: 806 207 bp). Subsequently Api m 6 gene-specific primers (GSP) for rapid amplification of cDNA ends (RACE; see further) were developed using the Primer3 software on the world wide web (Rozen & Skaletsky, 2000). This gave the following result: GSP forward primer (GSP-fw) 5′-TTGGAGGATTTGGAGGCTTGGAGGA-3′ and GSP reverse primer (GSP-rv) 5′-GCATTTAGATCGCGGAACGCATACCT-3′. Their suitability for further analysis was tested by a simple polymerase chain reaction (PCR) using the venom gland cDNA preparation (see above) as template. The resulting amplicon was sequenced for confirmation.

### 5′-Rapid amplification of cDNA ends (5′-RACE) and 3′-RACE

RACE ready cDNA was prepared by following the protocol described in the GeneRacer™ Kit (Invitrogen). Briefly, 100 ng of bee venom gland mRNA was subsequently treated with calf intestinal phosphatase and tobacco acid pyrophosphatase, to be ligated at its 5′-end with the GeneRacer™ RNA oligo. This ligated mRNA was reverse transcribed using the SuperScript™ III RT and the GeneRacer™ oligo dT primer to create RACE-ready first-strand cDNA with known priming sites at the 5′- and 3′-ends. Generation of 5′-RACE fragment was done by PCR, using a combination of GeneRacer™ 5′-primer and GSP-rv, whereas the 3′-amplification needed a combination of GSP-fw and GeneRacer™ 3′ primer. Both reactions were done in an EppeΛdorf Mastercycler using a touchdown protocol.

### Cloning and DNA sequencing

PCR products were cloned in the pCR®4-TOPO® vector. Individual transformants were picked and analysed for the presence of insert by PCR. The corresponding amplicons were used for sequence analysis.

DNA sequencing was performed using a Perkin Elmer ABI Prism 377 (Perkin Elmer, Wellesley, MA, USA) automated DNA sequencer. PCR product was treated with shrimp alkaline phosphatase (1 U/µl, Amersham E70092Y, Amersham Biosciences, Buckingham, UK) and exonuclease I (20 U/µl, Epicentre Biotechniologies ×40505k, Madison, WI, USA) for 15 min at 37 °C, followed by 15 min at 80 °C to inactivate the enzymes. This material was then used for cycle sequencing without any further purification, using the ABI Prism BigDye V 3.1 Terminator Cycle Sequencing kit. The sequencing conditions were 30 s at 96 °C, 15 s at 50 °C and 4 min at 60 °C for 27 cycles. Primers used for sequencing were GSP-fw, GSP-rv or GeneRacer™ 5′-primer. Cycle sequence products were precipitated by adding 25 µl of 95% ethanol and 1 µl 3 m sodium acetate, pH 4.6 to each cycle sequencing reaction (10 µl). The samples were placed at −20 °C for 15 min and centrifuged at 14 000 r.p.m. (12 225 *g*) for 15 min. After precipitation, an additional wash of the pellet was performed with 125 µl of 70% ethanol and centrifuged at 14 000 r.p.m. (12 225 *g*) for 5 min. The pellet was dried in a Speedvac concentrator, redissolved in loading buffer and run on a 48 cm 4.25% acrylamide/bisacrylamide (29 : 1) gel.

### Analysis of sequence data

Partial cDNA clones and the complete cDNA sequence generated above were aligned with honeybee genome assembly 4.0 (Amel_4.0–20061003) using BLASTN (Altschul *et al*., 1997), without filters. Sequences were also compared by BLASTN with unscaffolded contigs. Several thousand base pairs of flanking DNA on either side of Api m 6 (e.g. on Contig5539) was used to confirm that this contig and unassembled Contig14926 were in fact derived from the same genome location, despite showing substantial genome sequence variation at and near Api m 6. Unscaffolded Contig14926 was used, via BLASTN, to span the gap between assembled scaffolds 16.18 and 16.19. Sequence variation 2 5′- and 3′ UTR regions of Api m 6 was characterized by alignment of DQ384991 and Contigs14926 and 5539.
